# Supplementary material for: An Extensively Hydrolyzed Formula Supplemented with Two Human Milk Oligosaccharides Modifies the Fecal Microbiome and Metabolome in Infants with Cow’s Milk Protein Allergy
Source: Int J Mol Sci. 2023 Jul 13;24(14):11422. doi: 10.3390/ijms241411422 (PMC10379726; doi:10.3390/ijms241411422)
Supplement: Supplementary file 1 [file ijms-24-11422-s001.zip › 120723_CinnamonPaper_Supplementary_Figures_Tables_Methods_updated.pdf]

Supplementary Figures, Tables and Methods

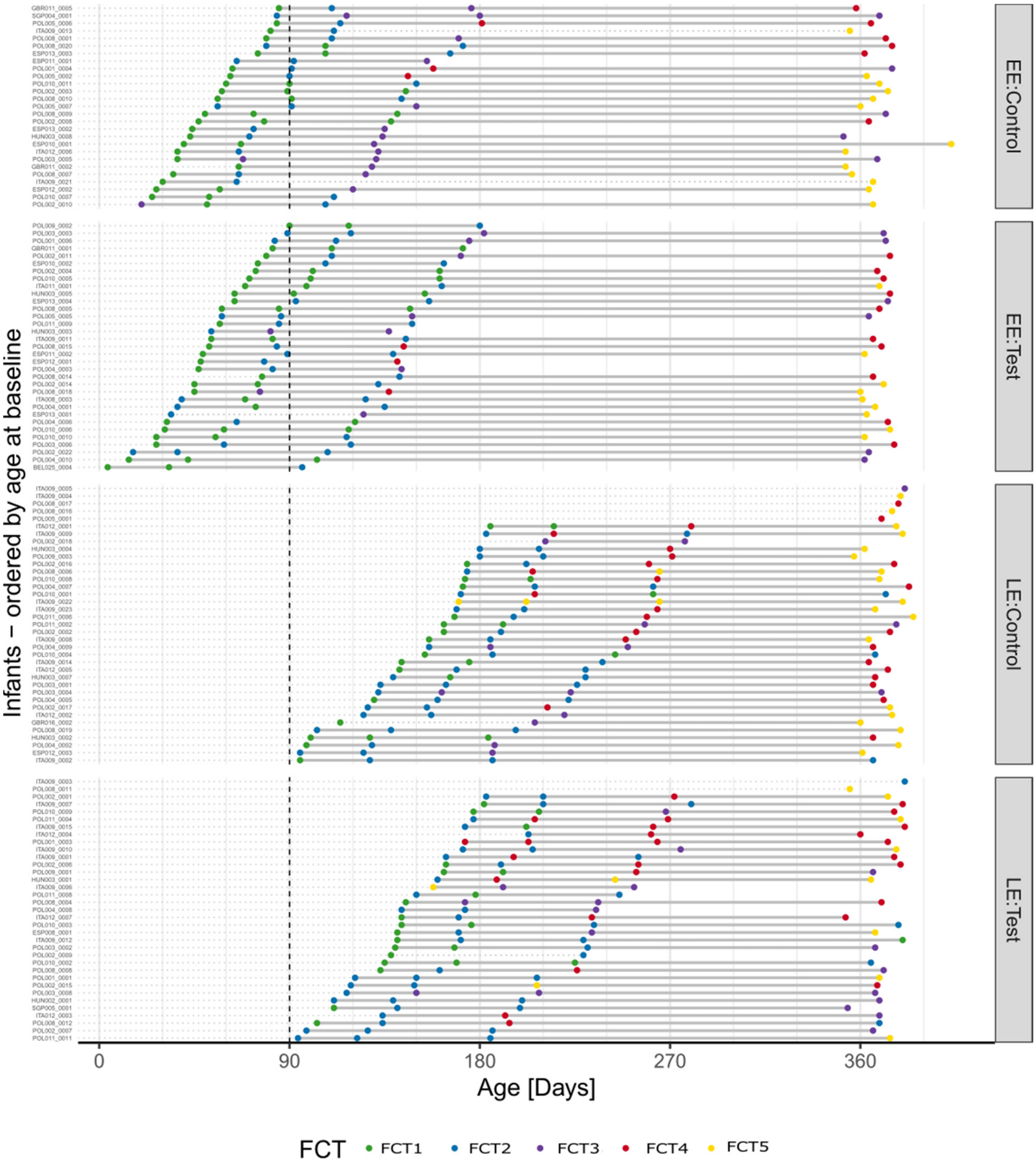

Supplementary Figure S1: Infant age and fecal community type (FCT) distribution across study. Dumbbell plot showing infant age and FCT distribution from baseline to 12 months of age for each feeding group in the early enrollment (EE) and late enrollment (LE) cohort. Each visit is represented by a dot. The FCT number at each visit is described by a color code.

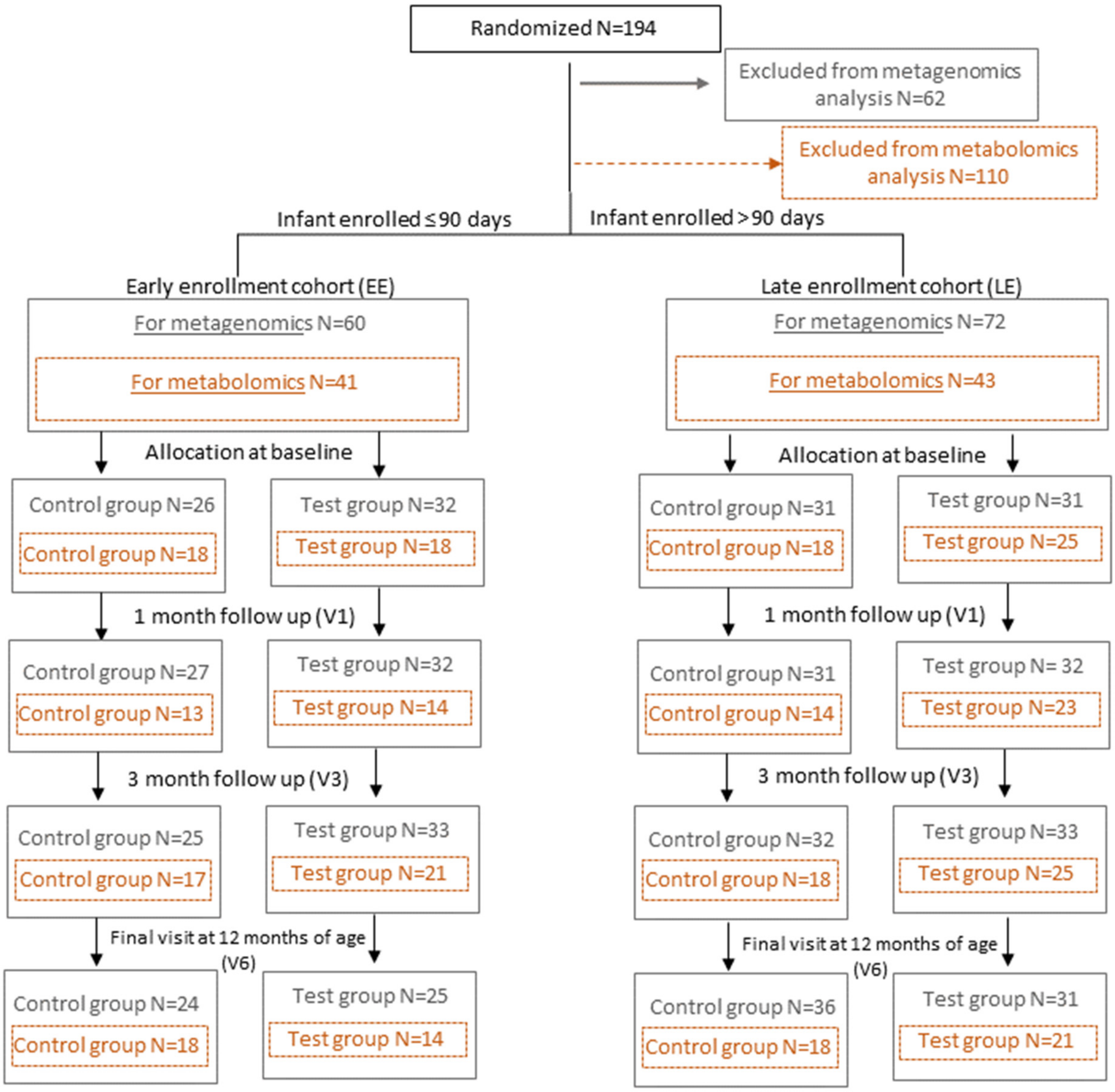

Supplementary Figure S2: Description of study participants. Number of infants in the control or test group at baseline, visit 1 (V1), visit 3 (V3) and visit 6 (V6) that were included for metagenomics and metabolomics. Due to the large age-heterogeneity at baseline, the cohort was stratified by early (EE; aged ≤90 days at baseline) and late enrollment LE >90 days at baseline). The reason for participant exclusion was insufficient sample volume to perform metagenomics or metabolomics analysis.

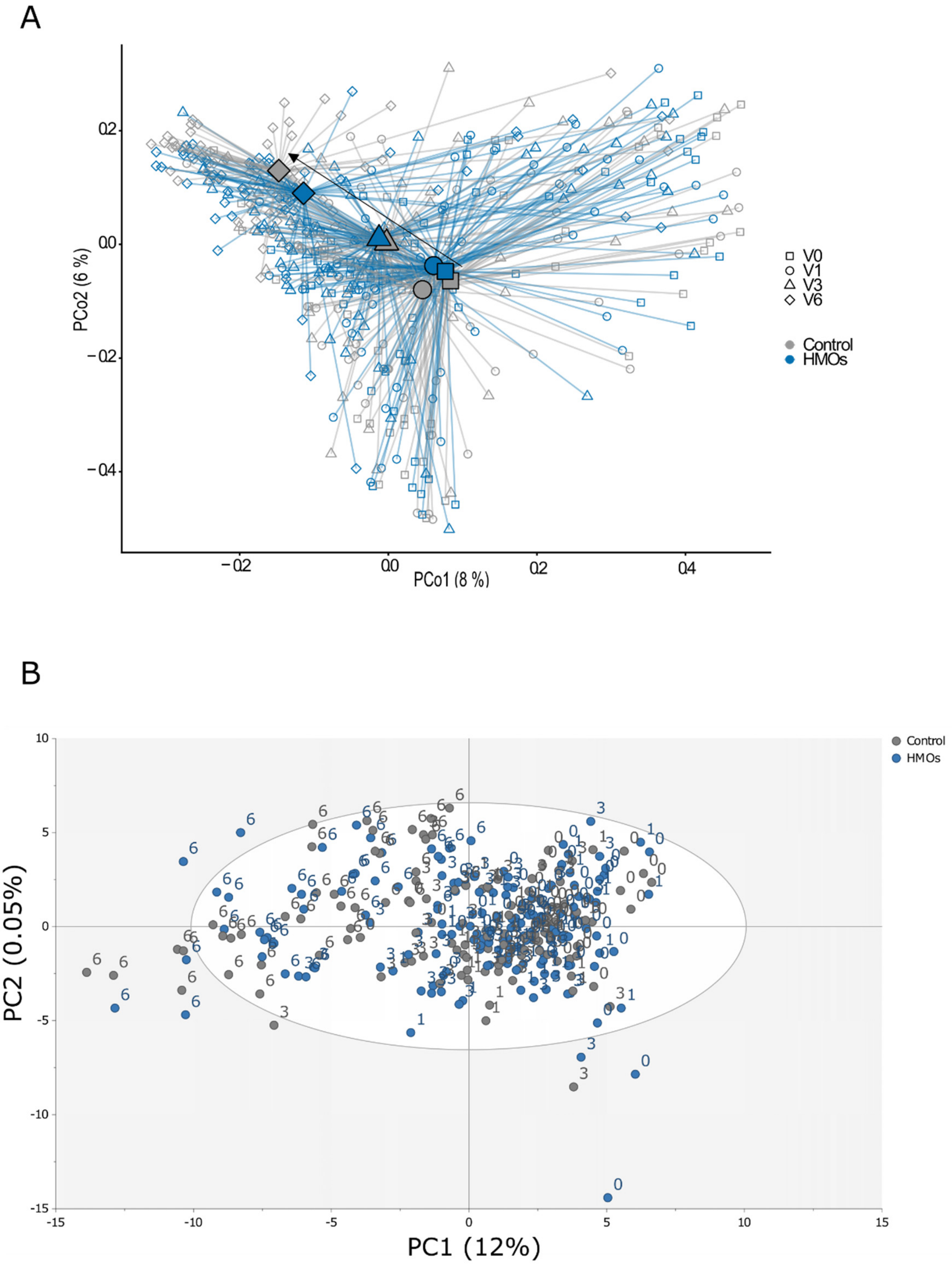

Supplementary Figure S3: Age gradient in fecal microbial and metabolomic signature. (A) PCoA based on Bray–Curtis dissimilarities among samples, calculated based on the MGS abundances. Each point represents a sample, and its color and shape indicate the treatment groups and visits, respectively. The mean (centroid) of samples in each group is indicated with a larger shape. Each sample is connected to its centroid by a thin line. The x- and y-axis labels

indicate the microbial variance explained by the first two principal coordinates. (B) PCA score plot showing the variance of metabolomics data explained by the first two components. Each point represents a sample, its color indicates the treatment group, and the labels indicate the visit number.

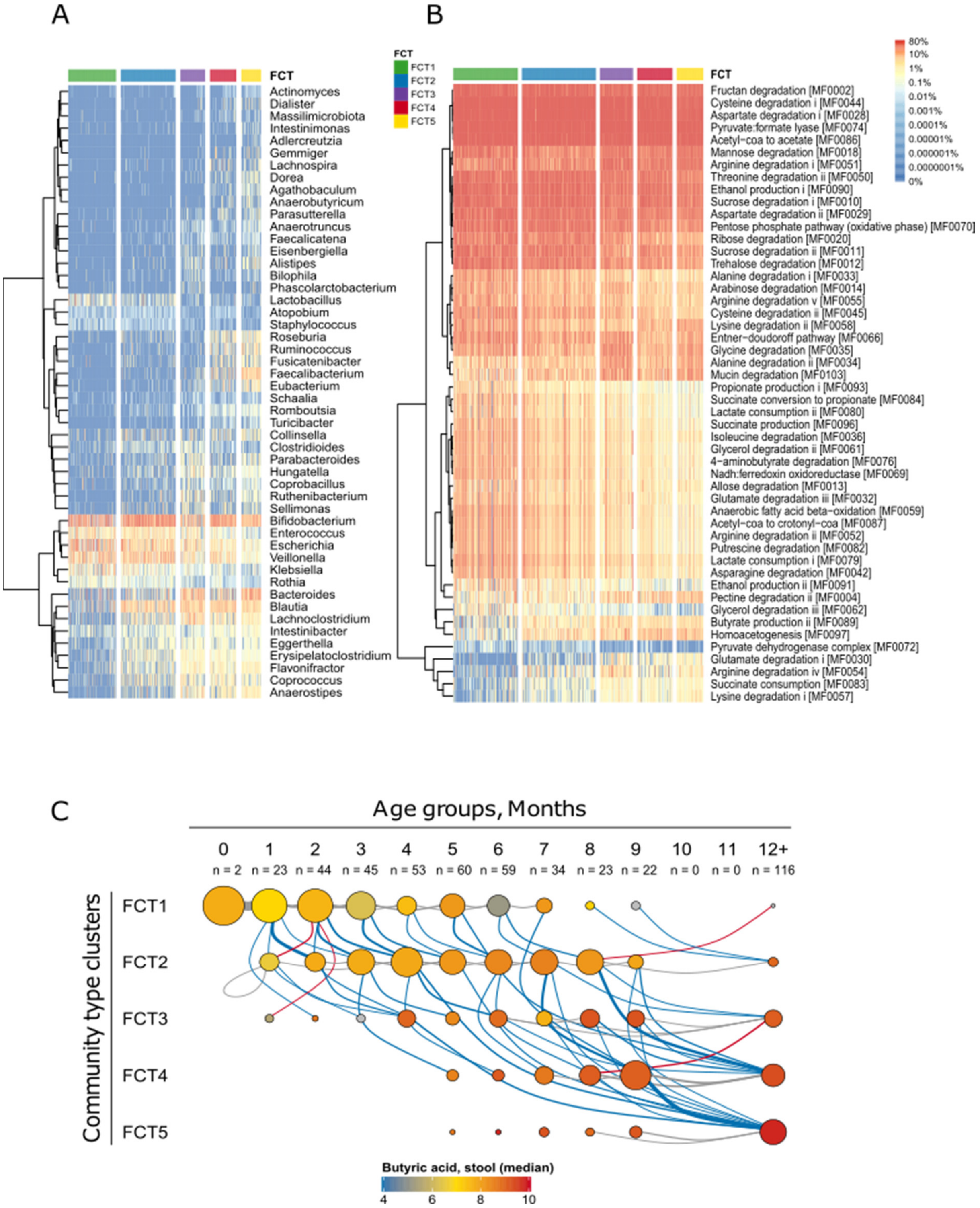

Supplementary Figure S4: **Summary characteristic of fecal community type** at genus level A) and B) functional level. The abundance was compared between the 5 FCT clusters using a Kruskal–Wallis test. The top 50 entities with the lowest p-values are shown in the heatmap. C) Transition model showing the progression of samples through each FCT. The trajectories are identical to Figure 2B, except that the nodes are colored based on the median concentration of butyric acid in stool for the samples with available metabolomics data assigned to the given FCT and age group. Grey node color indicates absence of stool metabolome data.

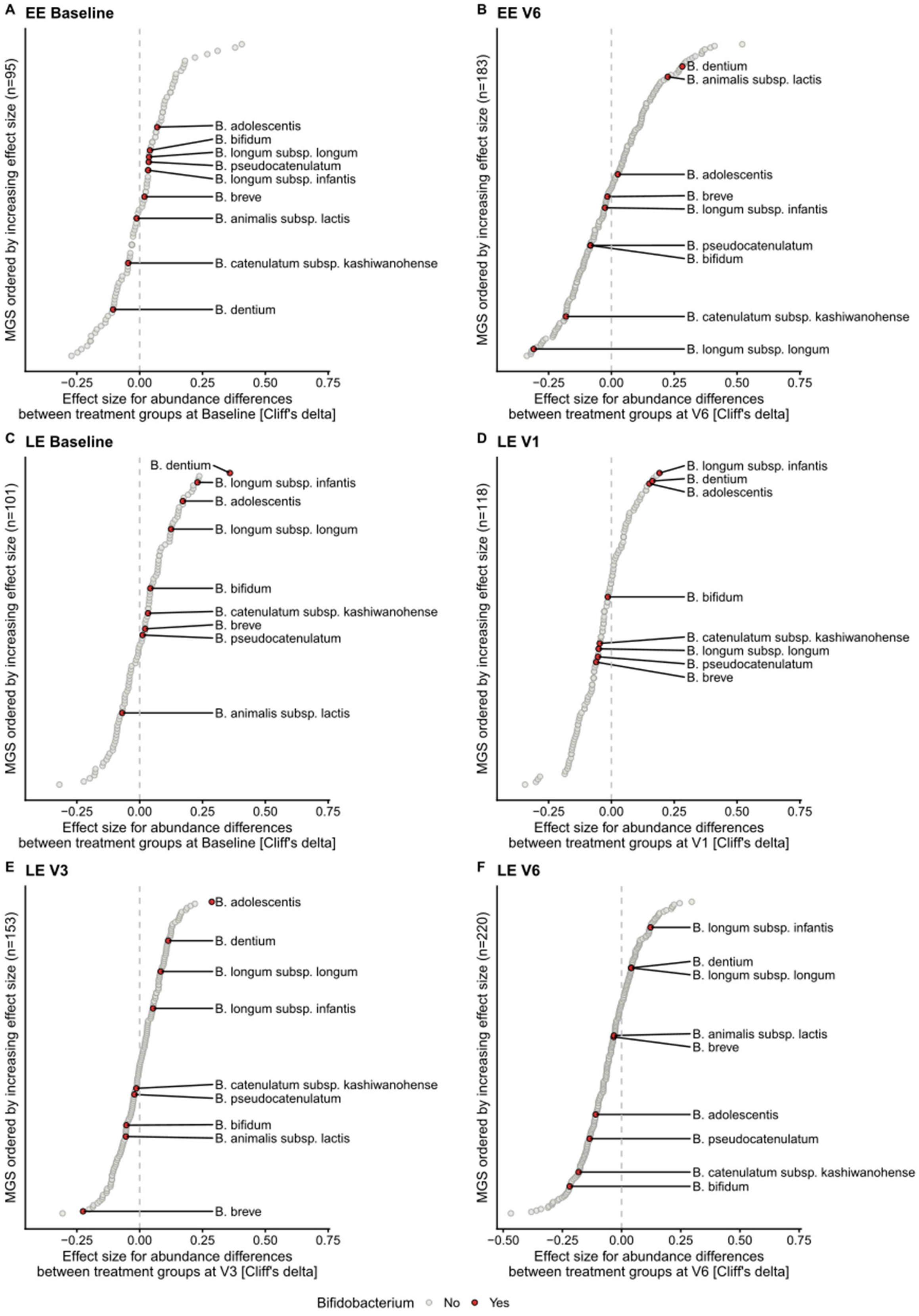

Supplementary Figure S5: Bifidobacterium species enrichment in Test group in EE and LE cohort. Each circle indicates the effect size (Cliff's delta) of a single metagenomic species (MGS) in comparison of relative abundance between the Control and Test groups at A)

baseline and B) V6 for the EE cohort; and C) baseline, D) V1, E) V3, and F) V6 in the LE cohort. The included MGS (y-axis) are sorted by their corresponding Cliff's delta (x-axis). For A, B the following number of samples were included At baseline/V6, EE Control, n=26/24; EE Test, n=32/25. For C, D, E, F, the following number of samples were included at baseline/V1/V3/V6: LE Control, n=26/27/25/24; LE Test, n=32/32/33/25. FCT: genus cluster, EE: Early enrolled, LE: Late enrolled.

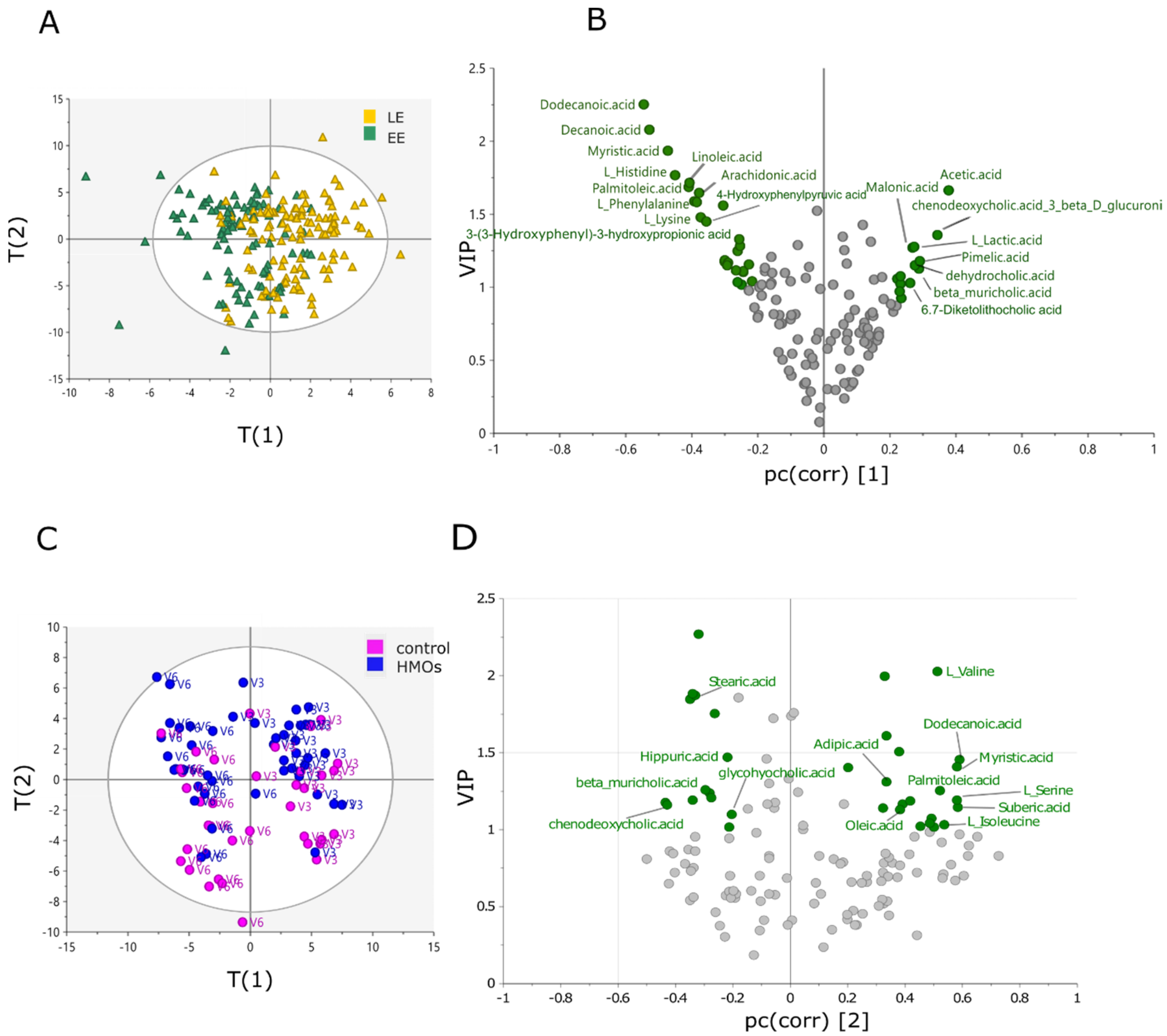

Supplementary Figure S6: **Metabolomics changes between cohorts and treatment effect s in LE cohort.** A,B) PLS-DA score plot modeling metabolomic changes between EE and LE cohorts after 1 month (V1), 3 month of treatment (V3) and 1 year of age (V6)(n=216 samples). A) The plot and statistics (supplementary Table 2) showed that the first component significantly discriminates LE from EE cohort. B) VIP plot showed cumulative VIP score and correlation coefficient to cohort (PC1). Most discriminating metabolites (VIP>1 & |PLS coefficient (p(corr))|>0.2) to feeding groups are highlighted in green. C) PLS regression score plot modeling age on the first component and feeding groups on the second component at V3 and V6 in the LE cohort (n=83 samples) D) VIP plot showing cumulative VIP score and correlation coefficient to treatment (PC2). Most discriminating metabolites (VIP>1 & |PLS coefficient (p(corr))|>0.2) to feeding groups are highlighted in green.

Supplementary Tables: See excel spreadsheet

Supplementary Table S1: Statistics of alpha diversity comparison (Faith's PD) between Test and Control groups when stratifying by visit (V0, V1, V3, V6) and by cohorts (EE, LE or all ages)
Supplementary Table S2: Statistics of FCT clusters distribution between Test and Control groups when stratifying by visit (V0, V1, V3, V6) and by cohorts (EE, LE or all ages).
Supplementary Table S3: Statistics of FCT cluster transition probabilities between Test and Control groups when stratifying by cohorts (EE, LE or all ages)
Supplementary Table S4: Statistics of Taxon Set Enrichment Analysis highlighting the taxonomic enrichment in Control and Test groups at family or genus level stratified by visit (V0, V1, V3, V6) and cohorts (EE, LE or all ages)**.** Positive Cliff's delta values imply the taxon is enriched in the Test group. Only Metagenomic species (MGSs) that had enough observations to be accepted with an FDR of 10% in a Wilcoxon Mann-Whitney test comparing the abundance of the test and control groups were considered. For each MGS that did not pass this threshold, N/A (not applicable) was included instead of the *p*-value.
Supplementary Table S5: List of metabolites measured with the targeted LC-MS method.
Supplementary Table S6: Statistics of PLS regression models discriminating age and treatment effects and OPLS-DA model discriminating LE and EE cohorts. Models were performed by combining samples from visits (V1, V3, V6) or visits at V3 and V6.
Supplementary Table S7: Summary of main metabolites discriminating Test and Control groups in the LE cohort at V3 and V6
Supplementary Table S8: Associations between selected KOs and treatment groups or FCTS stratified by visit (V3, V6) in the EE cohort. KEGG orthologues (KO) belonging to the amino acid or bile acid pathways were selected for the analysis.
Supplementary Table S9: Associations between metabolites and treatment groups or FCTS stratified by visit (V3, V6) in the EE cohort. Metabolites were selected if there were significantly different between control and test groups.
Supplementary Table S10: Inter domain correlations between selected metabolites and KEGG orthologues (KO) in the EE cohort.

Supplementary methods

Microbiome profiling

Fecal DNA extraction and sequencing

Microbial DNA was extracted and purified from frozen faeces using the NucleoSpin 96 Soil kit (Macherey-Nagel, Dueren, Germany). Lysis was performed by bead beating horizontally on a Vortex-Genie 2 at 2700 rpm for 5 minutes. Library preparation and next-generation sequencing were performed at Novogene (Cambridge, UK). The purified genomic DNA was randomly sheared into fragments of ~350 base pairs (bp) and used for library construction using the NEBNext Ultra Library Prep Kit for Illumina (New England Biolabs, Ipswich, USA). Libraries were evaluated using Qubit 2.0 fluorometer (Thermo Fisher Scientific, Waltham, USA) quantitation and Agilent 2100 Bioanalyzer (Agilent, Santa Clara, USA) for the fragment size distribution. Quantitative real-time PCR was used to determine the concentration of the final library prior to sequencing. Paired-end 2×150 bp sequencing was performed on an Illumina NovaSeq 6000 (Illumina, San Diego, USA).

Sequencing data processing

Quality control of the resulting FASTQ sequencing read files was performed using KneadData (v.0.6.1) to remove low-quality bases and reads derived from the host genome. Using Trimmomatic (v.0.36) [1], reads were quality trimmed by removing Nextera adapters, leading and trailing bases with a Phred score below 20 and trailing bases in which the Phred score over a window of size 4 dropped below 20. Trimmed reads shorter than 100 bases were discarded. Reads mapping to the human reference genome GRCh38 were discarded (Bowtie2 v. 2.3.2 at default settings[2]). Read pairs in which both reads passed filtering were retained.

Gene catalog and metagenomic species (MGS) definitions

The Clinical Microbiomics in-house infant fecal microbiome gene catalog (containing 23,968,023 microbial genes) was used for reference gene catalog and the corresponding set of 1306 metagenomics species (MGS) definitions for MGS abundance profiling. The MGSs were built based on >5000 deep-sequenced human adult and infant gut samples using an approach based on the metagenomic species concept [3] and have highly coherent abundance and base composition in a set of 1776 independent reference human gut samples.

Mapping reads to gene catalog

Trimmed, human-filtered reads were mapped to the gene catalog using BWA mem (v. 0.7.16a) [4]. PCR duplicates were removed using Samtools (v.1.6) [5]. An individual read was considered mapped to a gene if the mapping quality (MAPQ) was ≥ 20 and the read aligned with ≥ 95 % identity over ≥ 100 bp. However, if > 10 bases of the read did not align to the gene at either end, the read was considered unmapped. Reads meeting these criteria except for the MAPQ threshold were considered multi-mapped. Each read pair was counted as either 1) mapped to a specific gene, if both individual reads mapped to the same gene, or one read mapped to a gene and the other was unmapped, multi-mapped, or mapped to another gene in the same MGS, or 2) multi-mapped, if both reads were multi-mapped, or mapped to genes in different MGSs, or if one read was multi-mapped and the other one unmapped, or 3) unmapped, if neither individual read mapped. The resulting gene count table, of number of mapped read pairs for each gene, was used to calculate the relative abundance of each MGS.

Taxonomical annotation of MGSs

MGSs were annotated by blasting catalog genes to the NCBI RefSeq genome database (2018-10-01) using a minimum of 80% sequence coverage with varying levels of similarity: 95, 95, 85, 75, 65, 55, 50, and 45% for gene taxonomy annotation at subspecies, species, genus, family, order, class, phylum, and superkingdom level, respectively. To assign species, genus, family, order, class, and phylum or superkingdom level taxonomy to an MGS, we required 75, 60, 50, 40, 30, and 25%, respectively, of its genes to be consistently annotated to the same taxa at the given level. Furthermore, for species and at genus level annotation, we required that less than 10% of the remaining MGS genes to be annotated to any alternative taxon. Finally, we applied CheckM to each MGS [6], and updated our annotations with CheckM, annotation in cases where CheckM provided annotation at higher resolution (lower taxonomic rank).

MGS relative abundance calculation

For each MGS, a signature gene set had been previously defined, as the 100 genes optimized for accurate abundance profiling of the MGS. An MGS count table was created by counting the number of reads mapped to the MGS signature genes per sample. An MGS was considered detected if reads from a sample mapped to at least three of its signature genes; measurements that did not satisfy this criterion were set to zero. Based on internal benchmarks, this threshold results in 99.6% specificity. The MGS count table was normalized according to effective gene length and then normalized sample-wise to sum to 100 %, resulting in relative abundance estimates of each MGS.

All alpha diversity calculations was based on down sampled (rarefied) MGS abundance profiles to control for uneven sampling. These were calculated by random sampling, without replacement, of a fixed number of signature gene counts per sample, and then following the procedure described above.

Functional annotation and profiling

EggNOG-mapper software (v. 1.0.3, HMM mode) [7] was used to compare each gene in the gene catalog to the EggNOG (v. 4.5) orthologous groups database (http://eggnogdb.embl.de/), resulting in annotations for 65% of genes in the gene catalog. Genes with an EggNOG annotation were then mapped from EggNOG to the Kyoto Encyclopedia of Genes and Genomes (KEGG) orthology (KO) database (v. 78.2, https://www.genome.jp/kegg/) using MOCAT2 lookup tables (http://mocat.embl.de/).

Derivation of MGS-based species tree

The species tree for the MGS was created based on single-copy bacterial and archaeal marker genes from the Genome Taxonomy Database (GTDB) consisting of 120 bacterial and 122 archaeal marker-genes belonging to either TIGRFAM or PFAM protein families[8, 9]. First, INTERPROSCAN [10] was used to identify marker genes within each MGS. Multi-copy marker genes and marker genes that were identified in < 10 MGS were excluded, resulting in a total of 111 bacterial and 26 archaeal marker genes with sufficient coverage. 7 of the 130 marker genes were shared between bacteria and archaea. MGSs with fewer than 10 marker genes identified by this method and MGS that were annotated as eukaryotes were excluded. Protein sequences from these 130 marker genes were aligned using HMMalign (v.3.2.1), and non-aligned residues were trimmed from the multiple sequence alignment. The species tree was next inferred using the concatenation-based species tree approach in IQtree[11] with 1000 ultrafast bootstraps[12] and an edge-linked partition model[13]. The species tree covered a total of 1,255 MGS and was rooted with archaea as an outgroup.

Taxon set enrichment analysis

Taxon set enrichment analysis was performed at the family and genus level, as follows: Only MGSs that had enough observations to be accepted with an FDR of 10% in a Wilcoxon Mann-Whitney test comparing the abundance of the two treatment groups were considered. For each of these MGSs, a Wilcoxon Mann-Whitney test of relative abundances was performed, and MGSs were sorted on the effect size (Cliff's delta) of the test such that MGSs that are more abundant in one group are at the top of the list and vice versa.

Adequate statistical power was assured by only evaluating genera including at least three MGSs. For each remaining family or genus, perform a Wilcoxon Mann-Whitney test, comparing the ranks of MGSs belonging to the taxa to the ranks of all other MGSs. Any genera or family thus identified is considered significantly enriched among MGSs that are associated with the investigated contrast. The direction of the association (enrichment or depletion) depends on the Cliff's delta of the Wilcoxon Mann-Whitney test.

Chemicals and Reagents for Metabolomics profiling

All of the standards used were obtained from Sigma-Aldrich (St. Louis, MO, USA), Santa Cruz (Dallas, TX, USA), Steraloids Inc. (Newport, RI, USA) and TRC Chemicals (Toronto, ON, Canada). All the standards were accurately weighed and prepared in appropriate solution to obtain individual stock solution. Appropriate amount of each stock solution was mixed to create stock calibration solutions. Formic acid was of analytical grade and obtained from Sigma-Aldrich (St. Louis, MO, USA). Methanol (Optima LC-MS), acetonitrile (Optima LC-MS), and isopropanol (Optima LC-MS) were purchased from Thermo Fisher Scientific (Fair Lawn, NJ, USA). Ultrapure water was produced by a Milli-Q Reference system equipped with a LC-MS Pak filter (Millipore, Billerica, MA, USA).

References

1. Bolger, A.M., M. Lohse, and B. Usadel, *Trimmomatic: a flexible trimmer for Illumina sequence data.* Bioinformatics, 2014. **30**(15): p. 2114-20.
2. Langmead, B. and S.L. Salzberg, *Fast gapped-read alignment with Bowtie 2.* Nat Methods, 2012. **9**(4): p. 357-9.
3. Nielsen, H.B., et al., *Identification and assembly of genomes and genetic elements in complex metagenomic samples without using reference genomes.* Nat Biotechnol, 2014. **32**(8): p. 822-8.
4. Li, H. and R. Durbin, *Fast and accurate short read alignment with Burrows-Wheeler transform.* Bioinformatics, 2009. **25**(14): p. 1754-60.
5. Li, H., et al., *The Sequence Alignment/Map format and SAMtools.* Bioinformatics, 2009. **25**(16): p. 2078-9.
6. Parks, D.H., et al., *CheckM: assessing the quality of microbial genomes recovered from isolates, single cells, and metagenomes.* Genome Res, 2015. **25**(7): p. 1043-55.
7. Huerta-Cepas, J., et al., *Fast Genome-Wide Functional Annotation through Orthology Assignment by eggNOG-Mapper.* Mol Biol Evol, 2017. **34**(8): p. 2115-2122.
8. Parks, D.H., et al., *A complete domain-to-species taxonomy for Bacteria and Archaea.* Nat Biotechnol, 2020. **38**(9): p. 1079-1086.
9. Parks, D.H., et al., *A standardized bacterial taxonomy based on genome phylogeny substantially revises the tree of life.* Nat Biotechnol, 2018. **36**(10): p. 996-1004.

10. Jones, P., et al., *InterProScan 5: genome-scale protein function classification.* Bioinformatics, 2014. **30**(9): p. 1236-40.
11. Nguyen, L.T., et al., *IQ-TREE: a fast and effective stochastic algorithm for estimating maximum-likelihood phylogenies.* Mol Biol Evol, 2015. **32**(1): p. 268-74.
12. Hoang, D.T., et al., *UFBoot2: Improving the Ultrafast Bootstrap Approximation.* Mol Biol Evol, 2018. **35**(2): p. 518-522.
13. Chernomor, O., A. von Haeseler, and B.Q. Minh, *Terrace Aware Data Structure for Phylogenomic Inference from Supermatrices.* Syst Biol, 2016. **65**(6): p. 997-1008.
